# The local transcriptional regulators SacR1 and SacR2 act as repressors of fructooligosaccharides metabolism in *Lactobacillus plantarum*

**DOI:** 10.1186/s12934-020-01403-3

**Published:** 2020-08-10

**Authors:** Chen Chen, Linlin Wang, Haiyan Yu, Huaixiang Tian

**Affiliations:** School of Perfume and Aroma Technology, Shanghai Institute of Technology, Shanghai, 201418 People’s Republic of China

**Keywords:** *Lactobacillus plantarum*, Fructooligosaccharides, Local regulatory mechanism, Transcriptional factors binding sites, Regulatory network

## Abstract

**Background:**

In *Lactobacillus plantarum*, fructooligosaccharides (FOS) metabolism is controlled by both global and local regulatory mechanisms. Although catabolite control protein A has been identified as a global regulator of FOS metabolism, the functions of local regulators remain unclear. This study aimed to elucidate the roles of two local regulators, SacR1 and SacR2, in the regulation of FOS metabolism in *L. plantarum* both in vitro and in vivo.

**Results:**

The inactivation of *sacR1 and sacR2* affected the growth and production of metabolites for strains grown on FOS or glucose, respectively. A reverse transcription-quantitative PCR analysis of one wild-type and two mutant strains (*ΔsacR1* and *ΔsacR2*) of *L. plantarum* identified SacR1 and SacR2 as repressors of genes relevant to FOS metabolism in the absence of FOS, and these genes could be induced or derepressed by the addition of FOS. The analysis predicted four potential transcription factor binding sites (TFBSs) in the putative promoter regions of two FOS-related clusters. The binding of SacR1 and SacR2 to these TFBSs both in vitro and in vivo was verified using electrophoretic mobility shift assays and chromatin immunoprecipitation, respectively. A consensus sequence of WNNNNNAACGNNTTNNNNNW was deduced for the TFBSs of SacR1 and SacR2.

**Conclusion:**

Our results identified SacR1 and SacR2 as local repressors for FOS metabolism in *L. plantarum*. The regulation is achieved by the binding of SacR1 and SacR2 to TFBSs in the promoter regions of FOS-related clusters. The results provide new insights into the complex network regulating oligosaccharide metabolism by lactic acid bacteria. 
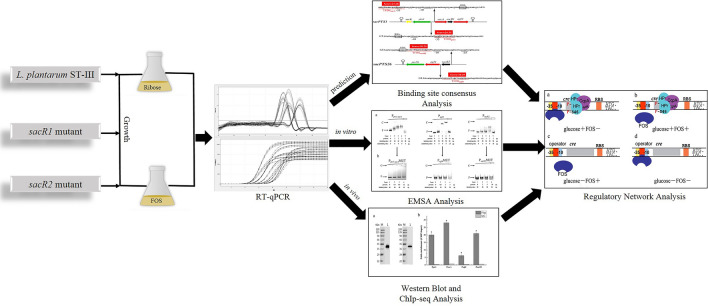

## Background

*Lactobacillus plantarum* is a Gram-positive bacterium that resides naturally in the human gastrointestinal tract (GIT) [1, 2]. This species is a common and versatile type of lactic acid bacteria (LAB) used in the production of several fermented and functional foods [1, 3–5]. Like most lactobacilli, *L. plantarum* strains have complex nutritional requirements for fermentable carbohydrates and can utilize a wide range of carbohydrates, including some prebiotics [4, 6, 7]. Fructooligosaccharides (FOS) are non-digestible food ingredients that can selectively stimulate the growth and activity of beneficial intestinal microbiota and are considered an established type of prebiotic [4, 8, 9]. Several studies have demonstrated that *L. plantarum* can effectively utilize FOS [10–12]. This advantage helps the survival and colonization of *L. plantarum* in the GIT [1, 6, 8].

In bacteria, the uptake and assimilation of different carbohydrates are tightly regulated, as the simultaneous utilization of all accessible sugars would be energetically inefficient [13]. The presence of preferred carbon sources prevents the utilization of secondary substrates via a phenomenon called carbon catabolite repression (CCR) [14, 15]. CCR, a complex regulatory phenomenon, is frequently mediated by several mechanisms [16] that either affect the synthesis of catabolic enzymes via global or specific regulators or inhibit the uptake of a carbon source and, consequently, the formation of the corresponding inducer [17]. According to previous reports, carbohydrate utilization by lactobacilli is always subject to CCR, which is achieved via the combined effects of global and operon-specific (i.e., local) regulatory mechanisms [18, 19]. Regarding the former type of regulatory mechanism, catabolite control protein A (CcpA) affects global transcriptional control by binding to catabolite repression element (*cre*) sites located in or downstream of the putative − 35 and − 10 sequences in the presence of more favorable carbon sources [20–24]. Regarding the latter, local regulons generally control a small number of genes and operons that are combined with specific operator motifs in the absence of the related substrate [25, 26]. Specifically, studies of the metabolic regulation of oligosaccharide utilization, such as FOS [27] and galactooligosaccharides (GOS) [28], have identified additional potential global and local regulatory factors in LAB, indicating that gene clusters associated with metabolic oligosaccharides are under the dual role of global and local regulation.

Previously, we identified two gene clusters, *sacPTS1* and *sacPTS26*, that are involved in the utilization of FOS in *L. plantarum* [11, 12]. The *sacPTS1* cluster is composed of five genes that encoded a sucrose phosphoenolpyruvate transport system (PTS1), a fructofuranosidase (SacA), a fructokinase (SacK), an α-glucosidase (Agl2), and a repressor (SacR1). The *sacPTS26* cluster encodes a sucrose PTS (PTS26), an α-glucosidase (Agl4), and a transcriptional regulator (SacR2) [11]. Specifically, two genes encoding the assumed repressor protein, *sacR1* and *sacR2*, were identified in the gene cluster associated with FOS metabolism and found to exhibit significant similarity to members of the GalR-LacI family of bacterial transcription regulators [11]. Subsequently, we demonstrated that CcpA is a vital regulator of FOS metabolism in *L. plantarum*, and it functions through the direct binding toward the *cre* sites in the promoter regions of FOS-related clusters [12]. However, the mechanism by which FOS metabolism is regulated via local regulators in *L. plantarum* remains unclear.

To determine whether FOS metabolism in *L. plantarum* is regulated locally by CCR, we firstly compared the physiological states of *L. plantarum* and mutant strains via growth profiles and metabolite production analysis. We then used reverse transcription-quantitative PCR (RT-qPCR) to compare the expression of relevant genes in cultures grown in chemically defined medium (CDM) with different sugars [29, 30]. Moreover, we predicted the presumed binding sites of local regulators in *L. plantarum* and verified these sites using electrophoretic mobility shift assays (EMSAs) and chromatin immunoprecipitation (ChIP) to detect in vitro and in vivo interactions, respectively. The results of this study shed new light on the network that regulates FOS metabolism in *L. plantarum* and reveals the essential roles of operon-specific transcriptional regulators in the control of FOS utilization.

## Results

### Growth profiles and metabolite production of the wild‑type and mutant strains

**Fig. 1:**
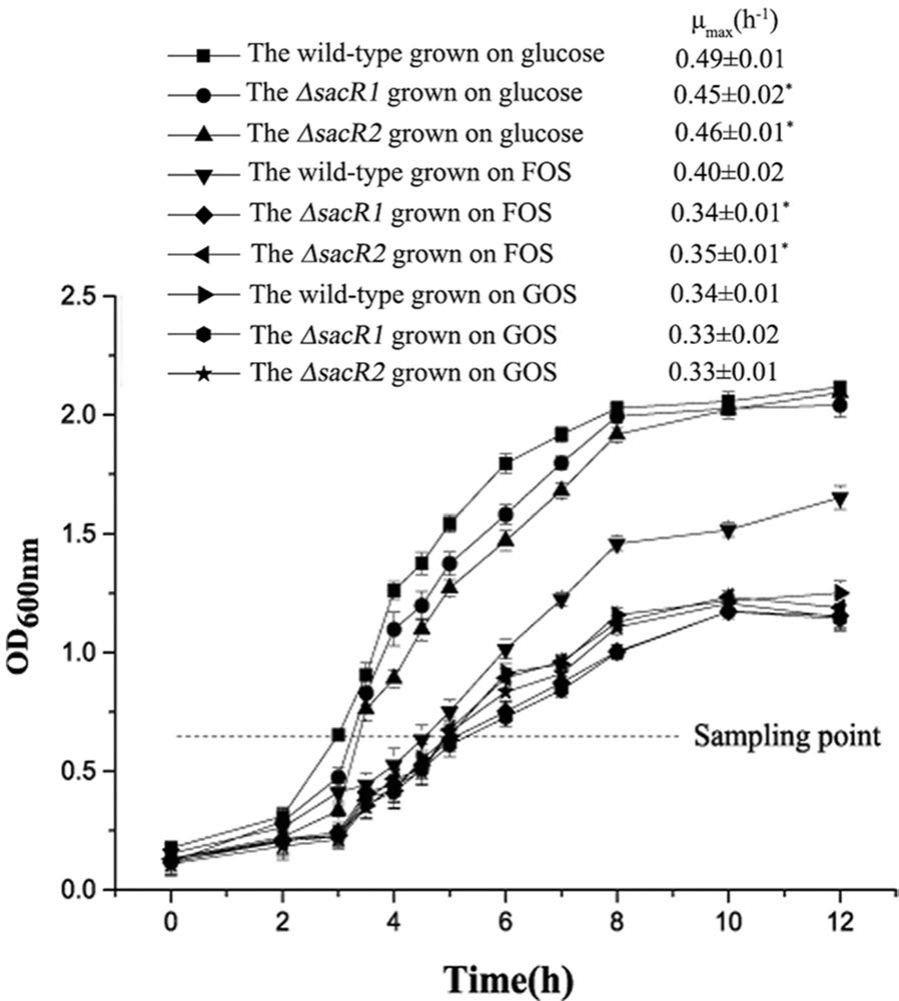
Growth curves of the wild-type and mutant strains (*ΔsacR1* and *ΔsacR2)* of *L. plantarum* ST-III in CDM containing glucose, FOS or GOS. Sampling point was chosen for the metabolite and RT-qPCR analysis. The for each condition was also calculated and shown in the figure. Data presented are mean values based on two replicate fermentations. Error bars indicate standard deviations. Asterisks indicate statistically significant differences (*P* < 0.05) of the corresponding μ_max_ values obtained from mutant strains compared with the wild-type

To determine the functions of SacR1 and SacR2 in FOS utilization, two mutant strains (*ΔsacR1* and *ΔsacR2*) were constructed using the Cre-lox-based mutagenesis system [31]. As the presence of glucose might induce global regulation [32, 33], which would mask the effect of local regulation [34]. We also selected another oligosaccharide—GOS that is not related to the FOS metabolism pathway for analysis and comparison [28, 35]. The growth of these mutant strains on glucose, FOS and GOS was compared with that of the wild-type strain (Fig. 1). The values of maximal growth rate (μ_max_) were significantly higher for the wild-type than the mutant strain (*P* < 0.05) in the logarithmic phase for glucose and FOS. For the strains grown on GOS, the growth curves were almost the same, and no difference was observed among their μ_max_ values (*P* > 0.05).

Our past findings clearly indicated that the metabolites of *L. plantarum* are mainly lactate, acetate and formate [10–12, 35]. Therefore, we also determined the levels of organic acids generated during fermentation with *L. plantarum* ST-III and two mutant strains on glucose, FOS or GOS, respectively (Table 1). Lactate and acetate are the main end products, resulting from the fermentation of the three carbon sources. Wild-type and the two mutant strains grown on FOS or GOS produced less lactate and more acetate than grown on glucose (P < 0.05). The results suggest that a shift from homolactic fermentation to mixed fermentation has occurred, which is consistent with our previous results [12, 35]. In the absence of SacR1 and SacR2, the metabolic products decreased compared with corresponding values for the wild-type strain grown on FOS (*P* < 0.05). This situation also occurred in the presence of glucose (*P* < 0.05), although to a lesser extent. The levels of metabolites did not vary between the wild-type and two mutant strains grown on GOS. These results are also in agreement with the growth profiles for the wild-type and two mutant strains. These data suggest that the inactivation of *sacR1 and sacR2* impairs the growth and the metabolite formation of *L. plantarum* in cultures containing FOS or glucose, respectively.

**Table 1:** Comparison of metabolites resulting from the fermentation of glucose, FOS and GOS by wild-type *L. plantarum* ST-III and *ΔsacR1* and *ΔsacR2* strains at OD_600_ of 0.65

	Wild-type strain			*ΔsacR1* strain			*ΔsacR2* strain		
	Lactate	Acetate	Formate	Lactate	Acetate	Formate	Lactate	Acetate	Formate
Glucose	15.60 ± 0.19	3.17 ± 0.06	1.64 ± 0.10	15.01 ± 0.07^#^	2.95 ± 0.02^#^	1.48 ± 0.05^#^	15.04 ± 0.04^#^	2.88 ± 0.02^#^	1.43 ± 0.01^#^
FOS^a^^,^^b^	12.34 ± 0.17*	5.63 ± 0.10*	1.47 ± 0.15*	11.09 ± 0.05*^,#^	4.35 ± 0.03*^,#^	1.23 ± 0.14*^,#^	10.90 ± 0.38*^,#^	4.21 ± 0.12*^,#^	1.26 ± 0.10*^,#^
GOS	12.28 ± 0.10*	6.08 ± 0.15*	1.49 ± 0.06*	12.19 ± 0.01*	6.26 ± 0.11*	1.46 ± 0.06	12.18 ± 0.39*	6.11 ± 0.15*	1.43 ± 0.21*

Data presented are mean values based on two replicate fermentations. Error bars indicate standard deviations

^a^Asterisks indicate statistically significant differences (*P *< 0.05) of the corresponding values obtained from cells grown on FOS or GOS compared with those grown on glucose

^b^Octothorpes indicate statistically significant differences (*P* < 0.05) of the corresponding values obtained from *ΔsacR1* or *ΔsacR2* mutant compared with those of wild-type

### RT-qPCR revealed repressor roles of SacR1 and SacR2

The differential expression of relevant genes was studied in comparison with growth on FOS and GOS through an RT-qPCR analysis. Our previous results showed that FOS is transported intact across the membrane by two PTSs (PTS1 and PTS26) and hydrolyzed by SacA into fructose and glucose-6-phosphate. Fructose is converted to fructose 6-phosphate under the action of SacK [11, 12]. Thus, the C_T_ values for these three genes, *sacK*, *sacA*, and *pts26*, were selected as the key genes for FOS metabolism and used to calculate the fold changes between conditions. As expected, the expression of all three genes was significantly up-regulated in the wild-type strain in the presence of FOS relative to GOS (*P* < 0.05, Table 2). In contrast, after *sacR1* and *sacR2* inactivation, the induction or derepression of the expression of these genes in response to FOS was nearly absent (*P* > 0.05, Table 2). For example, the levels of *sacA* and *sacK* expression in the wild-type strain exposed FOS were 2.62 and 3.01-fold higher, respectively, than those in wild-type cells exposed to GOS. In contract, the expression levels of these two genes were roughly the same in the presence of FOS and GOS to the *ΔsacR1* strain. Similar expression patterns were also observed for the *sacPTS26* operons and in the *ΔsacR2* strain. These results verify that SacR1 and SacR2 repress expression of genes relevant to FOS metabolism in the absence of FOS, FOS metabolism could be induced or derepressed by the addition of FOS.

**Table 2:** Relative transcript abundances of FOS-related genes in the wild-type and *ΔsacR1* and *ΔsacR2* strains grown in different sugars

Gene	Wild-type strain		*ΔsacR1* strain		*ΔsacR2* strain	
	FOS	GOS	FOS	GOS	FOS	GOS
*sacPTS26*^a,b^	3.10 ± 0.32*	1.51 ± 0.17	3.14 ± 0.18*	1.22 ± 0.05	3.04 ± 0.31	3.27 ± 0.16
*sacA*^a,b^	3.35 ± 0.29*	1.28 ± 0.23	3.18 ± 0.14	3.24 ± 0.24	3.42 ± 0.12*	1.11 ± 0.09
*sacK*^a,b^	3.16 ± 0.36*	1.05 ± 0.19	3.02 ± 0.22	3.08 ± 0.27	2.95 ± 0.31*	0.87 ± 0.14

Data presented are mean values based on at least three replicates. Error bars indicate standard deviations

^a^The relative transcription abundances of each gene in different conditions were calculated by the 2^−ΔCt^ method and 16S rRNA was used as the internal standard

^b^Asterisks indicate statistically significant differences (*P *< 0.05) of the corresponding values obtained from cells grown on FOS compared with those grown on GOS

### Analysis of binding site consensus

**Fig. 2:**
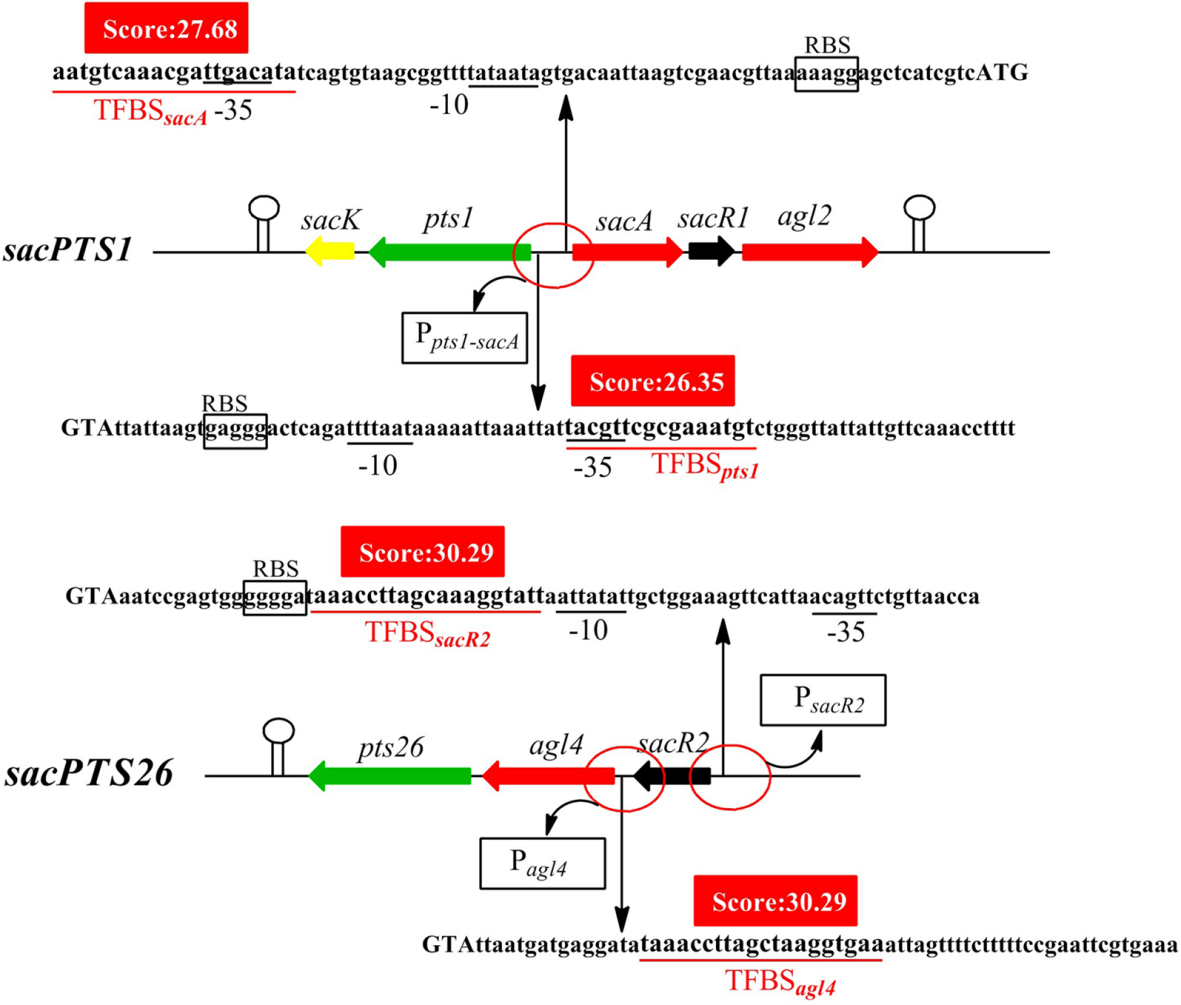
Predicted potential transcription factor binding sites (TFBSs) of SacR1 and SacR2 in the *sacPTS1* and *sacPTS26* clusters of *L. plantarum* ST-III. Putative TFBSs are underlined in red. The red backgrounds indicate the scores for each TFBS, defined as the sum of the positional nucleotide weight. The presumed start codon of each gene is shown in uppercase letters, and the putative -10 and -35 promoter regions and possible ribosome-binding sites (RBSs) are marked. P_*pts1-sacA*_, P_*agl4*_, P_*sacR2*_ in the black box represent the promoter regions

**Fig. 3:**
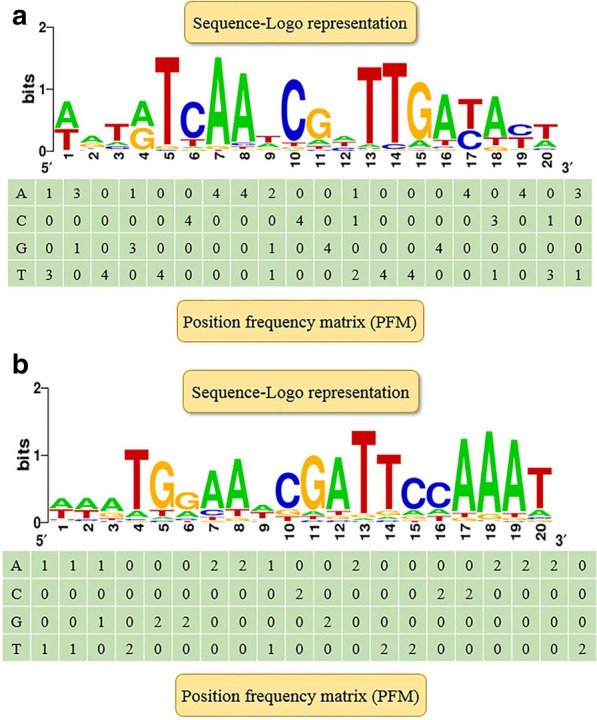
Consensus sequence motif of the transcription factor binding sites (TFBSs) in *L. plantarum* ST-III, generated using RSAT software. A positional frequency matrix (PFM) was generated according to the frequency of occurrence of each base at each location of the consensus sequence. The sequence-logo represents the occurrence frequency, and the height of each individual symbol reflects its prevalence at a given position. **a** Consensus sequence motif of the SacR1; **b** Consensus sequence motif of the SacR2

Local regulators can regulate target genes by interacting with specific transcription factor binding site (TFBS) in the operon [36, 37]. However, the binding sites used by SacR1 and SacR2 had not previously been elucidated. Accordingly, we searched the RegPrecise database for a conserved common binding consensus motif based on the profiles of TFBSs of local regulators in *L. plantarum* WCSF1. First, a positional frequency matrix (PFM) was constructed according to the frequency of occurrence of each base at each location of the consensus sequence (Fig. 2). Next, the generated PFM was used to search the *sacPTS1* and *sacPTS26* clusters, where two potential TFBSs were identified in the P_*pts1−sacA*_ region (TFBS-1, AATGTCAAACGATTGACATA; TFBS-2, TACGTTCGCGAAATGT). Additionally, one binding site each was identified in the P _*sacR-agl4*_ region (TFBS-3, TAAACCTTAGCTAAGGTGAA) and the P_*sacR*_ region (TFBS-4, AAACCTTAGCAAAGGTATT) (Fig. 3). The scores of these four candidate sites were all > 5, suggesting SacR1 and SacR2 binding [38].

### Confirmation of the binding of local regulators to sequence motifs in vitro

**Fig. 4:**
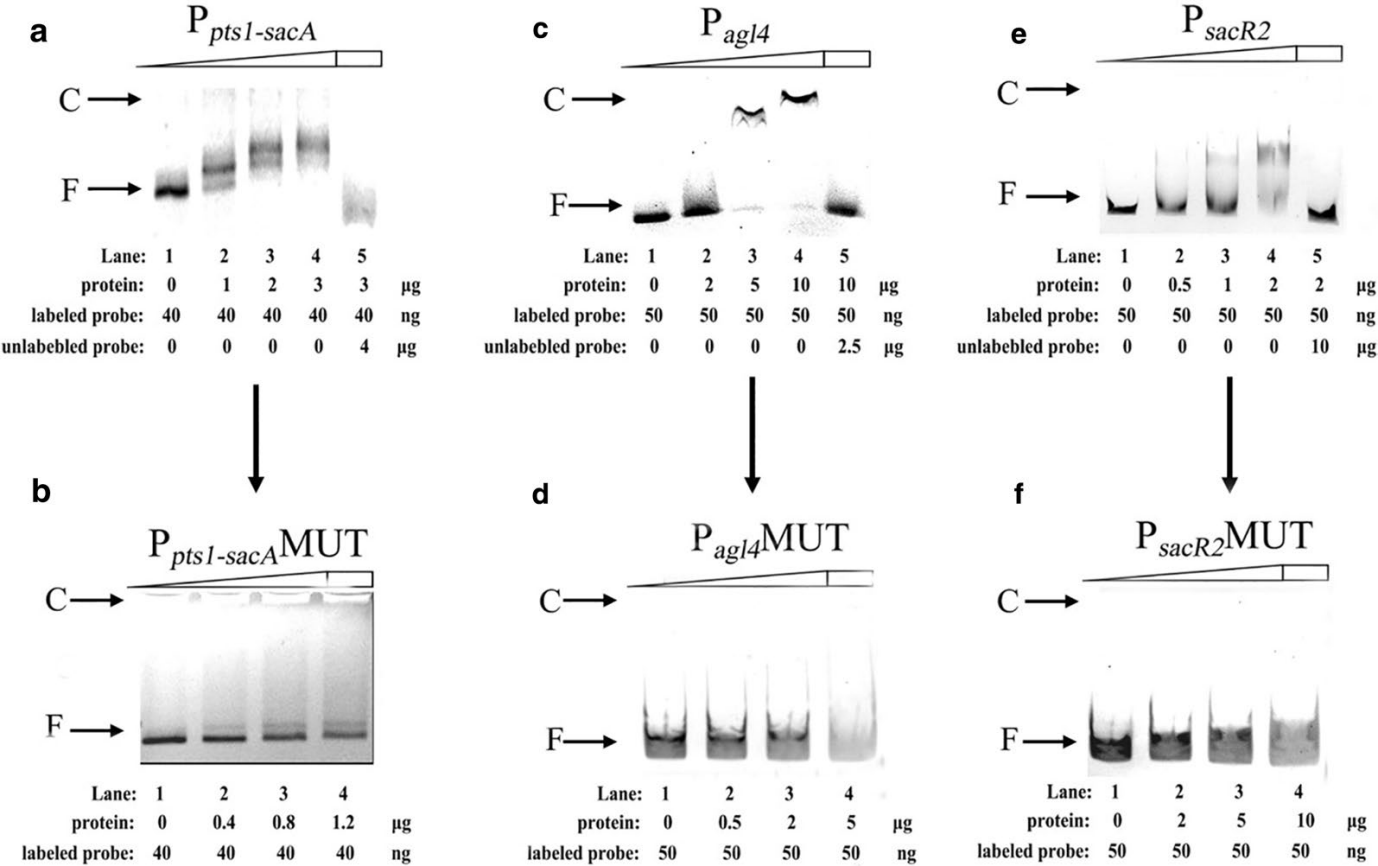
Characterization and verification of SacR1-DNA and SacR2-DNA binding at the four promoter regions by electrophoretic mobility shift assays (EMSAs). **a**, **b** The binding of His_6_-tagged SacR1 with DNA fragments of promoter region of P_*pts1-sacA*_ and its mutated site. **c**, **d** The binding of His_6_-tagged SacR2 with DNA fragments of promoter region of P_*agl4*_ and its mutated site. **e**, **f** The binding of His_6_-tagged SacR2 with DNA fragments of promoter region of P_*sacR2*_ and its mutated site. The positions of the SacR1-DNA and SacR2-DNA complexes (**c**) or free DNA (**f**) are indicated at the left of the figure

We next performed EMSAs to identify the four putative TFBSs to which the SacR1 and SacR2 proteins bind specifically in vitro [39, 40]. For the first step, both proteins were expressed successfully in different recombinant strains (BL21-*sacR1* and BL21-*sacR2*) and purified. Then the purified protein was used to perform EMSAs with the DNA probes generated in the possible promoter regions of the *sacPTS1* and *sacPTS26* clusters. As shown in Fig. 4a, c, e (lanes 1–4), the amounts of the SacR1-DNA and SacR2-DNA complexes increased with increasing concentrations of His_6_-tagged SacR1 (0–3 μg) and His_6_-tagged SacR2 (0–10 μg) proteins. In contrast, when labeled and unlabeled probes were used in a specific competitive assay (lane 5), no shift was detected for the labeled probe, indicating the binding specificities of SacR1 and SacR2 for these DNA fragments. Furthermore, except for the binding shown in Fig. 4, SacR1 cannot bind to the putative TFBSs of SacR2, and vice versa (data not shown).

Next, to verify the specific binding of SacR1 and SacR2 to the TFBSs, each putative TFBS which was generated from Regulatory Sequence Analysis Tools (RSAT) analysis according to the consensus motif, was mutated and named as TFBS-MUT. The main principle of the mutation was as follows: the defined base in the consensus motif was mutated to the other three bases, and the “W” that represents A or T was mutated to “S”, which represents G or C [33, 40]. DNA fragments of the three promoter regions containing the TFBS-MUT sites were generated by PCR and used in EMSAs [41]. Notably, the binding of His_6_-tagged SacR2 to the mutant P_*agl4*_ and P_*sacR2*_ regions was completely abolished (Fig. 4d, f). As the P_*pts1*−*sacA*_ region exists at two putative binding sites, it was mutated twice prior to the EMSA. The binding affinity of His_6_-tagged SacR1 protein for the P_*pts1*−*sacA*_ region was weakened after a single mutation (TFBS_*sacA1*_; data not shown). After the double mutation, His_6_-tagged SacR1 could no longer bind to the new P_*pts1*−*sacA*_ region (Fig. 4b). In conclusion, these results indicate that SacR1 and SacR2 proteins could bind specifically to the putative TFBSs in the *sacPTS1* and *sacPTS26* clusters, respectively.

### Confirmation of the binding of local regulators to the sequence motifs in vivo

**Fig. 5:**
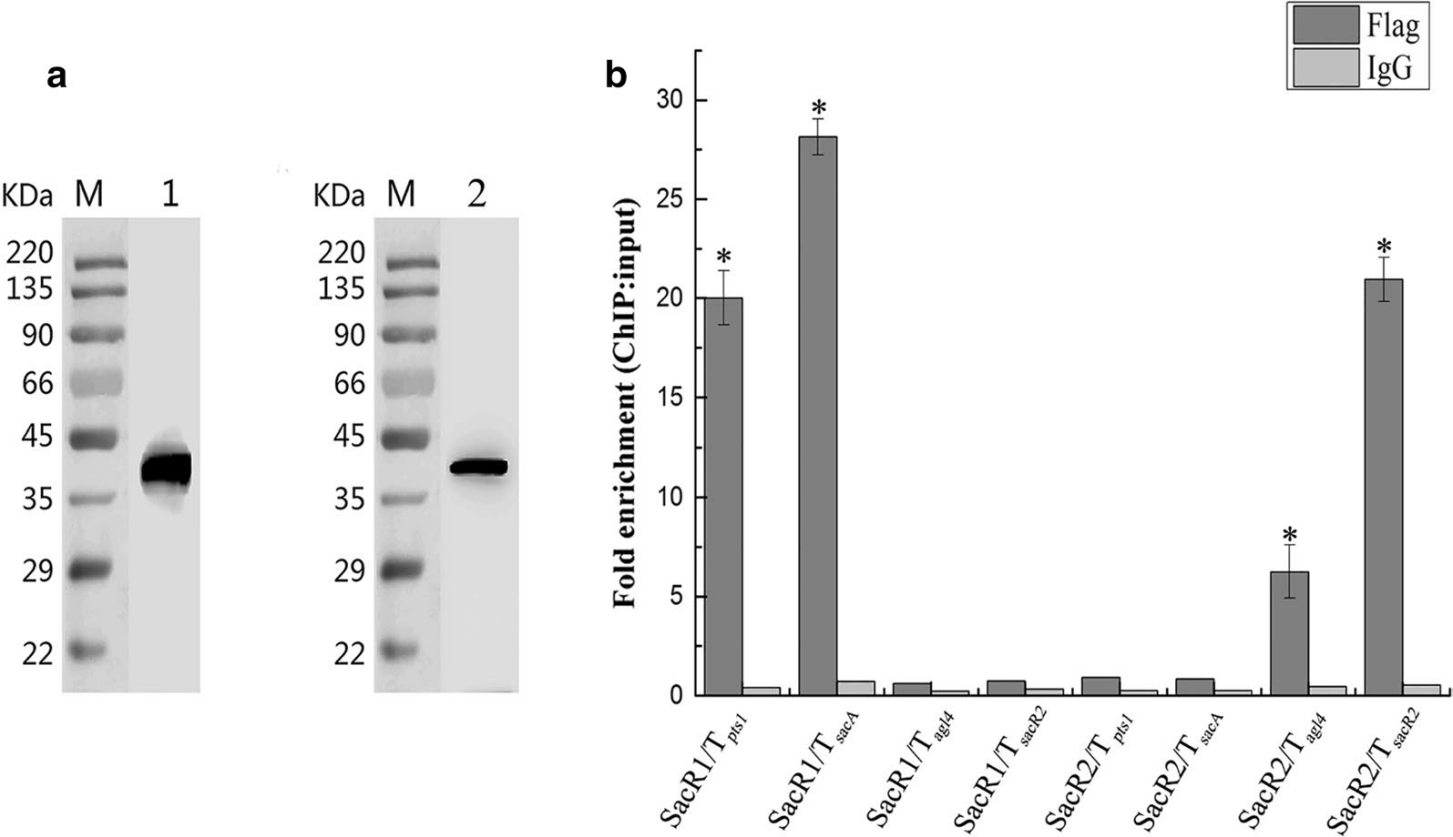
Chromatin immunoprecipitation (ChIP) analysis of the binding of SacR1 and SacR2 to the transcription factor binding sites (TFBSs) in the three promoter regions. **a** Detection of FLAG-tagged SacR1 and SacR2 proteins by western blotting with a FLAG-specific antibody. 1, SacR1; 2, SacR1. **b** Enrichment of FLAG-tagged SacR1 and SacR2 with TFBSs as determined by ChIP-qPCR. SacR1-T_*pts1*_, SacR1-T_*sacA*_, SacR1-T_*agl4*_, SacR1-T_*sacR2*_ represent the binding of SacR1 to the TFBSs and SacR2-T_*pts1*_, SacR2-T_*sacA*_, SacR2-T_*agl4*_, SacR2-T_*sacR2*_ represent the binding of SacR2 to the TFBSs. Data are presented as mean values based on at least three replicates. Error bars indicate standard deviations. Values that differed significantly (*P* < 0.05) from those of the negative control (normal rabbit IgG) are indicated with asterisks

Next, we performed a ChIP-qPCR analysis to validate the predicted interactions of SacR1 and SacR2 proteins with TFBSs in vivo. SacR1 and SacR2 were labeled with N-terminal FLAG-tags, and the subsequent successful expression of 409-Flag-*sac*R1 and 409-Flag-*sac*R2 in *L. plantarum* was confirmed via a western blot analysis (Fig. 5a). Next, both the ChIP-extracted and input DNA were examined by qPCR. As shown in Fig. 5b, the fragments TFBS_*pts1*_ and TFBS_*sacA*_ were remarkably enriched (22.0 and 28.1-fold, respectively) by IP with the FLAG-tagged SacR1 protein when compared with mock ChIP samples, demonstrating that SacR1 interacts specifically with the P_*pts1*−*sacA*_ region in vivo. Similarly, the fragments TFBS_*agl4*_ and TFBS_*sacR2*_ were also remarkably enriched (6.2- and 20.9-fold, respectively) by IP with FLAG-tagged SacR2. No other enrichment was observed in Fig. 5, which once again proved that SacR1 cannot bind to the TFBSs of SacR2, as is the case for SacR2. Together, these findings suggest that SacR1 and SacR2 can bind specifically to their corresponding TFBSs in the identified promoter regions of the two clusters.

## Discussion

*L. plantarum* is a versatile species that can grow on numerous types of carbohydrates. Notably, this bacterial species can utilize FOS, although in a relatively low efficiency compared with glucose, and harbors two gene clusters that participate in FOS metabolism [11]. Due to the complex interspecies competition in the GIT, the regulation of FOS metabolism is crucial to the survival and colonization of *L. plantarum*. We have verified that CcpA, a GalR-LacI family protein, is a vital regulator of FOS metabolism in *L. plantarum*; two local regulators, SacR1 and SacR2, are involved in the regulation of these two FOS metabolism-related clusters [12]. These results suggested that the utilization of FOS in *L. plantarum* may involve the double effects of global and local regulation. However, the specific manner by which SacR1 and SacR2 control local regulation have not been determined. In this report, we evaluated the regulation of FOS metabolism by local regulatory elements in *L. plantarum* both in vitro and in vivo.

The CCR in response to glucose may have been predominant in the context of dual regulation, whereas the effects of local regulators could not be observed [18]. Thus, we also included GOS as an alternative carbon source to verify the roles of SacR1 and SacR2. Combining the results of growth profiles, metabolite production and gene expression, we found that inactivation of these two local regulators significantly affected growth and fermentation end-products in *L. plantarum* and they acted as repressors of FOS-related genes in the absence of FOS. The deletion of *sacR1* and *sacR2* could also affect the growth and metabolite formation for strains grown on glucose, but to a lesser extent. These two regulators did not affect the metabolism of GOS. These results revealed the regulatory complexity for sugar utilization in *L. plantarum*.

The regulation of locally regulated gene transcription involves the binding of specific regulators to binding sites on the target genes [25, 26]. However, potential SacR1 and SacR2 binding sequences had not previously been clarified in the two FOS metabolism-related clusters in *L. plantarum*. In this study, we identified four putative TFBSs in the promoter regions of FOS-related clusters. These sites were predicted based on the consensus motif generated from RSAT analysis. Then the specific binding interactions in vitro and in vivo were verified in this study by EMSA and ChIP-qPCR, respectively. Although both regulators showed a low level of sequence identity (28%), they both belong to the GalR-LacI family of CcpA-like proteins and are expected to have similar DNA-binding features [11, 26]. When combining the predicted TFBSs of SacR1 and SacR2 with our present results, we deduced a consensus sequence for the SacR1 and SacR2 binding sites, WNNNNNAACGNNTTNNNNNW (N = any base, W = A or T), which is also similar to the consensus sequence of *cre* sites [19, 26]. However, no cross reaction was observed for SacR1 and SacR2 with other TFBSs. A possible reason is that although TFBSs of SacR1 and SacR2 are similar in structure, some differences in the sequences may hinder the binding by other regulators. These results provide a new insight into the structures of local regulator recognition sites in Gram-positive bacteria. Related foot-printing and CHIP-seq experiments to confirm the binding of SacR1 and SacR2 to the target sites are ongoing.

**Fig. 6:**
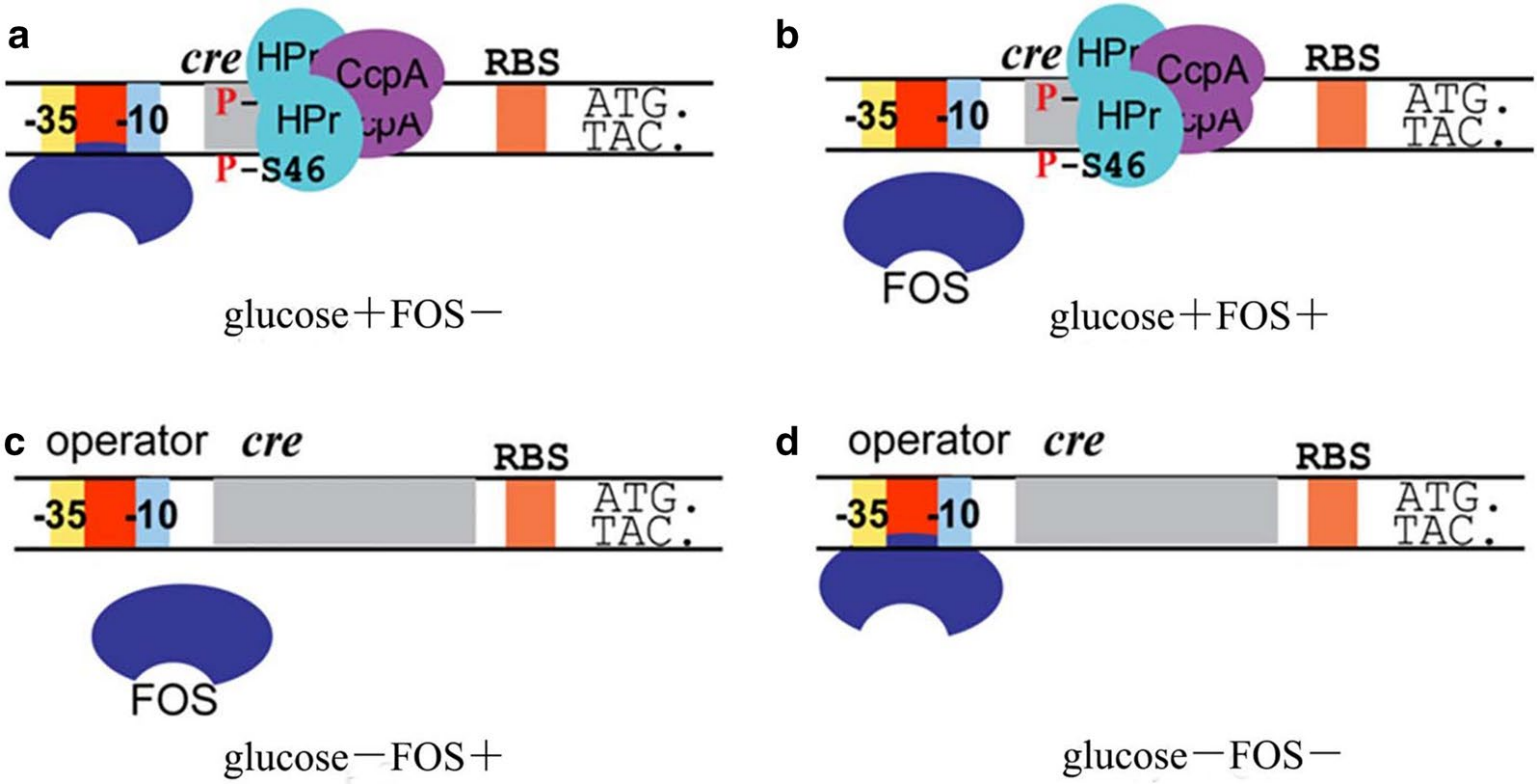
Mechanisms underlying the global and local regulation of FOS metabolism in *L. plantarum*. **a** Presence of glucose. **b** Presence of both glucose and FOS. **c** Presence of FOS. **d** Absence of glucose and FOS

Many studies revealed a double effect of global and local regulation on carbohydrate metabolism in LAB [42, 43]. In contrast to these global regulators, local regulators regulate only one or a few genes that are often linked genetically to the gene encoding the regulator itself [44]. For instance, Tamara et al. [45] identified a novel RpiR-family transcription activator, GlaR, positioned directly upstream of the *gal-lac* gene cluster in *Lactococcus lactis* IL1403. GlaR was identified as a transcriptional activator of galactose and lactose utilization genes, the expression of which can be induced by galactose. Moreover, six LacI-family local transcriptional factors and a TetR-family regulator were identified as presumptive local repressors of arabino-oligosaccharide (AOS) utilization in *Bifidobacterium* species [46]. According to our previous studies and the present work, FOS metabolism is regulated both globally and locally in *L. plantarum* [11, 12]. Regulation can be divided into four conditions based on the available carbon source, as follows: only glucose, only FOS, both glucose and FOS, and neither glucose nor FOS. These conditions enable the deduction of the possible regulatory mode. If only glucose is present (Fig. 6a), the binding of CcpA to *cre* sites would block the transcription of FOS-related genes in *L. plantarum*. If only FOS is present (Fig. 6c), FOS would bind to repressor proteins (SacR1 and SacR2) to reverse the inhibition induced by the binding of SacR1 and SacR2 to TFBSs in the promoter regions of FOS-related clusters. If neither source is present (Fig. 6d), SacR1 and SacR2 act as repressors and inhibit the expression of FOS-related clusters. If both sources are present (Fig. 6b), FOS acts as an inducer, thus rendering the repressor proteins allosteric and releasing inhibition; however, the global regulator CcpA binds to *cre* sites and thus remains capable of eliciting CcpA-mediated CCR. This latter process is also the cause of the diauxic growth phenomenon, in which cells resume growing and enter a second growth phase fueled by FOS as the carbon source once glucose is depleted [12]. However, the actual mechanism of regulation may be more complex, as these local regulators are also activated or repressed by CcpA [10, 12]. Furthermore, SacR1 and SacR2 are co-transcribed with other FOS-related genes, suggesting that both proteins act as self-regulators to maintain their own expression [10]. In summary, FOS metabolism is an extremely complex network in which the combined actions of global and local regulators orchestrate the transcription of various units that enable bacteria to adjust sugar utilization to their metabolic capacities.

## Conclusions

In summary, we performed a systematic study of the local regulation of FOS metabolism in *L. plantarum*. The inactivation of *sacR1 and sacR2* impaired the growth of *L. plantarum* and the metabolite formation in cultures containing FOS or glucose, respectively. The RT-qPCR data demonstrated SacR1 and SacR2 inhibited the expression of genes relevant to FOS metabolism in the absence of FOS, and these genes could be induced or derepressed by FOS. Furthermore, we predicted four potential TFBSs for SacR1 and SacR2 binding in multiple regions of the two FOS-related clusters in *L. plantarum*. We then verified the direct binding of SacR1 and SacR2 to these TFBSs in vitro and in vivo by EMSA and ChIP, respectively, which suggests that SacR1 and SacR2 act as local regulators through direct regulation of the transcription of FOS-related clusters. As local regulation is a component of FOS metabolism in *L. plantarum*, a further analysis of global and local regulation may give us a deeper understanding of the complex regulatory network underlying this metabolism. This information would serve as a theoretical basis upon which to construct an overall regulatory network of oligosaccharide metabolism by *L. plantarum *in vivo and could be also used as a model to study the utilization of oligosaccharides for other LAB.

## Methods

### Bacterial strains, plasmids, oligonucleotides, and culture conditions

The strains and plasmids used in the present study are summarized in Table 3. The oligonucleotide primers used are listed in Additional file 1: Table S1. *Escherichia coli* (*E. coli*) DH5α and BL21, which were used for the cloning and/or expression of genes of interest, were propagated in Luria Bertani (LB) broth at 37 °C with aeration at 200 rpm/min. *L. plantarum* ST-III and its mutant strains were cultivated anaerobically in deMan–Rogosa–Sharpe (MRS) broth (Merck, Darmstadt, Germany) at 37 °C without aeration. Where appropriate, the culture medium was supplemented with antibiotics at the following concentrations. To select antibiotic-resistant strains of *E. coli*, 100 μg/mL kanamycin, 50 μg/mL ampicillin, 30 μg/mL chloramphenicol, and 250 μg/mL erythromycin were added to LB. To select mutant strains of *L. plantarum*, 10 μg/mL chloramphenicol and 10 or 30 μg/mL (for replica plating) erythromycin were added to MRS medium.

**Table 3:** Strains and plasmids used in this study

Strain and plasmid	Relevant feature	Source or reference
Strains		
*L. plantarum*		CGMCC 0847
ST-III	Wild type	
* ΔsacR1*::cat	Derivative of ST-III containing a *lox66*-P32-*cat*-*lox71* replacement of *sacR1*	This study
* ΔsacR2*::cat	Derivative of ST-III containing a *lox66*-P32-*cat*-*lox71* replacement of *sacR2*	This study
* ΔsacR1*	Derivative of ST-III containing a *lox72* replacement of *sacR1*	This study
* ΔsacR2*	Derivative of ST-III containing a *lox72* replacement of *sacR2*	This study
409-Flag-*sacR1*	Derivative of ST-III harboring pSIP409-Flag-*sacR1*	This study
409-Flag-*sacR2*	Derivative of ST-III harboring pSIP409-Flag-*sacR2*	This study
*E. coli*		
DH5α	For general gene cloning and plasmid construction	Promega
BL21	For protein expression	Novagen
BL21-*sacR1*	*E. coli* BL21 (DE3) harboring pTolo-EX5-*sacR1*	This study
BL21-*sacR2*	*E. coli* BL21 (DE3) harboring Pet28a-*sacR2*	This study
Plasmid		
pTolo-EX5	Ap^R^, for cloning and protein expression, included His-tag	Tolobio
pET-28a ( +)	Kana^R^, for cloning and protein expression, included His-tag	Novagen
pTolo-EX5-*sacR1*	Ap^R^, pTolo-EX5 with *sacR1* gene cloned into *Xho*I sites	This study
pET-28-*sacR2*	Kana^R^, pET-28a ( +) with *sacR2* gene cloned into *Nhe*I/*Hin*dШ sites	This study
pNZ5319	Cm^R^, Em^R^; for multiple gene replacements in Gram-positive bacteria	[29]
pNZ5319-up-down-1	Cm^R^, Em^R^; pNZ5319 derivative containing homologous regions up and downstream of *sacR1*	This study
pNZ5319-up-down-2	Cm^R^, Em^R^; pNZ5319 derivative containing homologous regions up and downstream of *sacR2*	This study
pNZ5348	Em^R^; contains *cre* under the control of the lp_1144 promoter	[29]
pSIP409	Em^R^; for shuttle vector in *E.coil*, *gusA* controlled by P_*sppQ*_	[53]
pSIP409-Flag-*sacR1*	Em^R^; pSIP409 derivative; *gusA* replaced by Flag-tagged *sacR1*	This study
pSIP409-Flag-*sacR2*	Em^R^; pSIP409 derivative; *gusA* replaced by Flag-tagged *sacR2*	This study

*Kana*^*R*^ kanamycin resistant; *Ap*^*R*^ ampicillin resistant, *Cm*^*R*^ chloramphenicol resistant, *Em*^*R*^ erythromycin resistant

### Construction of *sacR1* and *sacR2* mutants

The *L. plantarum* ST-III deletion strain was generated using the Cre-lox-based mutagenesis system [31]. The upstream and downstream DNA regions of *sacR1* and *sacR2* were amplified using the respective primer pairs (Additional file 1: Table S1). The resultant DNA fragments were cloned into the suicide vector pNZ5319 to yield the pNZ5319-up-down-1 and pNZ5319-up-down-2 plasmid constructs. These deletion plasmids were transfected into *L. plantarum* ST-III cells via electroporation, and deletion mutants were screened as described previously [12, 34]. Candidate double-crossover clones were confirmed by PCR analysis.

### Growth analysis and detection of metabolites under different carbon sources

For growth analysis, overnight cultures of *L. plantarum* ST-III and two deletion strains (*ΔsacR1* and *ΔsacR2*) were transferred with 2% (v/v) inoculum into 500 mL of CDM supplemented with filter-sterilized solutions of 1% (w/v) glucose, FOS (Meiji Seika Kaisha, Tokyo, Japan) or GOS (QuantumHi-Tech Biological Co., Ltd., Guangdong, China). The cultures were incubated for 16–18 h at 37 °C in a bioreactor (Bioflo model 115, New Brunswick Scientific Co., Edison, NJ, USA) and flushed with sterile air (0.1 v/v min), without agitation and controlling the value of pH. During the cells’ growth up to the stationary phase, the samples were withdrawn every 2 h to measure the optical density at 600 nm (OD_600_) for growth analysis. The values of μ_max_ were calculated through linear regressions of the plots of ln (OD_600_) versus time during the exponential growth phase [35]. When the OD_600_ reached 0.65 (early logarithmic phase), cultures were harvested by centrifugation (8,000 × *g*, 10 min, 4 °C). The supernatants were filtered through a 0.22 μm nylon filter (Titan, China). The production of main metabolites was analyzed by high-performance liquid chromatography (HPLC) respectively, as previously reported [12].

### RNA extraction and RT-qPCR analysis

FOS and GOS were selected as carbon sources for RNA extraction. The cultures were prepared as mentioned above and total RNA was extracted from exponentially growing wild-type and mutant cells (OD_600_ of 0.65) using TRIzol reagent (Invitrogen, Shanghai, China), as described previously [12]. Total RNA was then incubated with RNase-free DNase I and purified using a PrimeScript RT reagent kit (Takara Bio, Dalian, China). The quality and quantity of the RNA were evaluated using a Thermo Scientific Nanodrop 2000 device (Thermo, Waltham, MA, U.S.A.) and an Agilent 2100 Bioanalyzer (Agilent, Palo Alto, CA, U.S.A.), respectively.

For the RT-qPCR analysis, single-stranded cDNA was synthesized from total RNA using PrimeScript reverse transcriptase (Takara Bio, Dalian, China) according to the standard protocol. This synthesized cDNA was then used as a template for quantitative RT-PCR analysis, as described previously [10]. The primers used for the analysis are listed in Additional file 1: Table S1. All reactions were performed on the 7300 Fast Real-Time PCR System (Applied Biosystems, U.S.A.) using previously reported PCR cycling conditions [35]. To standardize the results, the relative abundance of 16S rRNA [47] was used as the internal standard, and the relative gene expression data were calculated and analyzed using the 2^−ΔCt^ method [48].

### Prediction of the binding sites of SacR1 and SacR2

RSAT was used to analyze the consensus motif of the TFBSs for SacR1 and SacR2. The motifs were identified by scanning all upstream regions in the genome of *L. plantarum* ST-III based on the profiles of gene binding sites (Lp_0188 and Lp_3221*)* in *L. plantarum* WCSF1 via the RegPrecise database [2]. A PFM was constructed to collect TFBSs, and putative TFBSs in the upstream regions of sacPTS1 and sacPTS26 clusters were searched. The scores of candidate sites were calculated as the sums of the positional nucleotide weights, as previously described [49], and values > 5 were considered indicative of potential TFBSs of SacR1 and SacR2.

### Purification of SacR1 and SacR2 proteins expressed in *E. coli*

Expression of the *sacR1* gene to produce recombinant protein was performed using the pTolo-EX5 vector (TOLO Biotech, Shanghai, China). Briefly, a 981 bp sequence of the *sacR1* gene was PCR amplified using the primer pair sacR1-F and sacR1-R, which includes the same *Xho*I site at the 5′ end of the primers (Additional file 1: Table S1). Subsequently, the amplified DNA was digested by *Xho*I and inserted into the corresponding site of the pTolo-EX5 vector. A 1,002 bp sequence of the *sacR2* gene was PCR amplified using the primer pair sacR2-F and sacR2-R, which include the *Nhe*I and *Hind*III sites at the 5′ end of the primers, respectively (Additional file 1: Table S1). Expression of the *sacR2* gene was achieved by digesting amplified DNA using the two restriction endonucleases, followed by insertion into the corresponding sites of the pET-28a ( +) expression vector. The resulting plasmids, pTolo-EX5-sacR1 and pET-28a-sacR2, contained the target gene fused to an N-terminal His-tag sequence. The recombinant plasmids were transformed as described previously [50], and the strain harboring these plasmids were named *E. coli* BL21- sacR1 and *E. coli* BL21- sacR2.

*E. coli* BL21(DE3) cells transformed with the two recombinant plasmids were grown at 37 °C in 100 mL of LB medium supplemented with kanamycin (150 μg /mL). When the OD_600_ reached 0.4–0.6, expression of the recombinant gene was induced by the addition of 1 mM isopropyl-b-D-thioisopropyl-b-D-thiogalactoside (IPTG). After an 8 h incubation at 25 °C, the cells were harvested by centrifugation. The His_6_-tagged proteins were extracted and purified by nickel ion affinity chromatography on a Chelating Sepharose Fast Flow column (GE Healthcare, Waukesha, WI, U.S.A.) according to the manufacturer’s instructions. The purified proteins were desalted and concentrated using Amicon Ultra-0.5 centrifugal filter devices (Millipore, Billerica, MA, U.S.A.). The resultant proteins were used in EMSAs.

### Electrophoretic mobility-shift assay (EMSA)

EMSAs were performed using 1 nM double-stranded DNA fragments (P*pts1* − s*acA*, P*agl4*, and P*sacR2*, ~ 200 bp) that were generated by PCR using specific primer pairs (Additional file 1: Table S1). The DNA fragments were located in the four promoter regions of the *sacPTS1* and *sacPTS26* clusters. The DNA probes were incubated with increasing quantities of the selected proteins in binding buffer (50 mM Tris–HCl, pH 8.0; 100 mM KCl; 2.5 mM MgCl_2_, 0.2 mM dithiothreitol [DTT]; 2 μg polydIdC; 10% [v/v] glycerol) in a total reaction volume of 20 μL for 30 min at 30 °C. The samples were loaded onto 2% agarose gels containing 0.5 × Tris–borate-EDTA buffer (TBE). To verify the specific binding of SacR1 and SacR2 to the TFBSs, each putative TFBS generated from the RSAT analysis according to the consensus motif was mutated and named TFBS-MUT (Additional file 1: Table S2). The mutations were introduced as previously reported [12].

### Chromatin immunoprecipitation assay (ChIP)

The respective *sacR1*and *sacR2* overexpression plasmids pSIP409-Flag-sacR1 and pSIP409-Flag-sacR2 were constructed by inserting the purified *sacR1* or *sacR2* coding sequence into a restriction enzyme-digested pSIP409 vector as described previously (Additional file 1: Table S1) [50]. Next, the recombinant plasmids were electroporated into *L. plantarum* ST-III, which were used to produce 409-Flag-*sacR1* and 409-Flag-*sacR2* for ChIP.

The ChIP procedure was modified from existing protocols [12]. Briefly, for the strains 409-Flag-*sacR1* and 409-Flag-*sacR2*, the cells were cultured at an OD_600_ of 0.3 and then induced with peptide pheromone IP-673 (synthesized by Invitrogen, Shanghai, China) in a final concentration of 50 ng/mL and allowed to grow for 2 h at 37 °C. Subsequently, in vivo cross-linking in the cultures was performed using 1% (v/v) formaldehyde for 20 min, and subsequently quenched by the addition of glycine to a final concentration of 0.125 M at room temperature for 5 min. The bacterial cells were collected by centrifugation at 5000 × g and 4 °C for 5 min and washed twice with ice-cold 5 mM Tris–HCl (pH 8.0). The pellet was resuspended in 5 mM Tris–HCl (pH 8.0) containing 5 μL of protease inhibitors. Bacterial chromatin was sheared by ultrasonic disintegration (Bioraptor plus, Diagenode, Belgium) for 5 min at 4 °C with input setting 6. After centrifugation, 5 mL of supernatant were transferred to a fresh tube as the input sample, and the remaining supernatant was added to the FLAG-binding beads overnight at 4 °C on a rotating wheel. On the next day, the beads were removed from the supernatant via magnetic separation (DynaMag™-2, Invitrogen, UK). The beads were washed four times in wash buffer (500 mM EDTA, 5 M NaCl, 1 M Tri-HCl, pH 8.0) and resuspended in 200 μL of elution buffer. The resulting supernatant was collected after magnetic bead separation, mixed with 5 M NaCl, and heated to 65 °C for 12 h to reverse cross-links. DNA was purified via phenol:chloroform extraction and ethanol precipitation [51]. The purified DNA samples were analyzed by qPCR using specific primers (Additional file 1: Table S1). Normal rabbit IgG was used as a negative control. All qPCRs were performed on the 7300 Fast Real-Time PCR System (Applied Biosystems) using a three-step PCR procedure (initial denaturation at 95 °C for 30 s, followed by 40 cycles of denaturation at 95 °C for 5 s, annealing at 54 °C for 25 s, and synthesis at 60 °C for 25 s). Product specificity was confirmed by a melting curve analysis. The qPCR results of each ChIP sample were normalized to a region of the 16S rRNA gene. Relative target levels were calculated using the fold enrichment method [52]. The results are reported as the average enrichment for three biological replicates.

### Statistical analysis

The data shown herein are representative of at least three independent experiments. Student’s t-test was used to determine statistical differences. Differences between values with P < 0.05 were considered statistically significant.

## Supplementary information

**Additional file 1: Table S1** Primers used in this study. **Table S2** Nucleotide sequences of oligonucleotides harboring the putative transcription factor binding sites (TFBSs) and mutated sites used for electrophoretic mobility shift assays (EMSAs).

## Data Availability

All data generated or analyzed during this study are included in this article and its additional file.

## Abbreviations

FOS: Fructooligosaccharides
TFBS: Transcription factor binding site
GIT: Gastrointestinal tract
LAB: Lactic acid bacteria
CCR: Carbon catabolite repression
CcpA: Catabolite control protein A
*cre*: Catabolite responsive elements
GOS: Galactooligosaccharides
RT-qPCR: Reverse transcription-quantitative PCR
CDM: Chemically defined medium
EMSA: Electrophoretic mobility shift assay
ChIP: Chromatin immunoprecipitation
μ_max_: Maximal growth rate
PFM: Positional frequency matrix
RSAT: Regulatory Sequence Analysis Tools
AOS: Arabino-oligosaccharide
*E. coli*: *Escherichia coli*
LB: Luria Bertani
MRS: De Man–Rogosa–Sharpe
OD: Optical density
HPLC: High-performance liquid chromatography
IPTG: Isopropyl-b-D-thioisopropyl-b-D-thiogalactoside
TBE: Tris–borate-EDTA buffer
