# Supplementary material for: The local transcriptional regulators SacR1 and SacR2 act as repressors of fructooligosaccharides metabolism in Lactobacillus plantarum
Source: Microb Cell Fact. 2020 Aug 10;19:161. doi: 10.1186/s12934-020-01403-3 (PMC7419226; doi:10.1186/s12934-020-01403-3)
Supplement: Supplementary file 1 — Additional file 1: Table S1 Primers used in this study. Table S2 Nucleotide sequences of oligonucleotides harboring the putative transcription factor binding sites (TFBSs) and mutated sites used for electrophoretic mobility shift assays (EMSAs). [file 12934_2020_1403_MOESM1_ESM.docx]

**Table S1 Primers used in this study.**

| Primer | Sequence (5’ to 3’) | |
| --- | --- | --- |
| **Primers for gene cloning** | | |
| sacR1F | GGAATTCCATATGATACTGGAGTCGATAACAATGAAACCCC | |
| sacR1R | CCGCTCGAGTTACGCGGTAAGGCCCTTG | |
| sacR2F | GGAATTCCATATGGCCTAAATGACAACGATTAGTGAAAT | |
| sacR2R | CCGCTCGAGGGCTTAAGCACTTTTTCTTTTGAT | |
| **Primers for gene mutation** | | |
| sacR1UpF | AGGACCGATAACGCGCTCGAGACGACTACTGAGATCGGTGCTATCT | |
| sacR1UpR | CGGTAGATTTAAATTGTTTAAACGCCGCGAAAACTTTTTCTTTAG | |
| sacR2UpF | CCGCTCGAGTGACGATTTATCAATTCAATTGGCC | |
| sacR2UpF | GCGTTTAAACAATCGCTGCCTGAATCTGTTTCTTC | |
| sacR1DownF | GCGAATTGTACACTGGAATCAAA | |
| sacR1DownR | GGAAGATCTTAGCGCCACTAACGGTGTCTT | |
| sacR2DownF | CATTTGCGGCAGTGGCCC | |
| sacR2DownR | GGAAGATCTAAGTTGGTCATAACTAACCCCCGAA | |
| sacR1-85 | GGTATACGAGTTCGTTTCGTGAT | |
| sacR1-87 | AGGAATCATTACCGAAGTAATCGT | |
| sacR1-108 | TAATGCCGACTGTACTTTCGG | |
| sacR1-109 | CATCATATCTGCCGGTGATAGTAG | |
| sacR2-85 | GTTTTTTTCTAGTCCAAGCTCACA | |
| sacR2-87 | TAATGCCGACTGTACTTTCGG | |
| sacR2-108 | TGGTTAGTACCACTCATATTTGGAA | |
| sacR2-109 | TAACTAACCCCCGAAGCCTC | |
| 120 | AGAACAATCAAAGCGAGAATAAGG | |
| 20 | AATAGTTATCTATTATTTAACGGGAGG | |
| CmF | TCTTAGTGACAAGGGTGATAAACTC | |
| CmR | CCGAACCATTATATTTCTCTACATC | |
| EmF | CGATACCGTTTACGAAATTGG | |
| EmR | CTTGCTCATAAGTAACGGTAC | |
| sacK1F | TTTAATGGTCGCACTCATACTGAAC | |
| sacK1R | CCAGCAGCTAAACCTTCTAAACAG | |
| sacAF | GGATGGACACGGATAATCACATT | |
| sacAR | TTGAGAAATAAACCAAGATAGCACC | |
| sacPTS2F | TTAACCAGTGGGATAGGTGCTG | |
| sacPTS2R | CGAGACGACCGTATAAATCAGC | |
| 16SF | CGCAAGGCTGAAACTCAAAGG | |
| 16SR | CTGACGACAACCATGCACCAC | |
| **Primers for target analysis and EMSA** | | |
| pts1F | AACGACTCAAAGTCACAAATGTCC | |
| sacAR | CAATCAGAGCATACTGGAGTCGAT | |
| agl4F | CAACCGGGGTAACATTTGGAT | |
| agl4R | GGCGGTCGATCAGCTATTACAT | |
| sacR2F | GTGGACTAAATGTTTTAAGGGCAAAC | |
| sacR2R | TGCCTGAATCTGTTTCTTCTTAGCA | |
| sacAmutR | GGGCTGTACCGTTCGTATAGCATAC | |
| pts1mut2F | TAGGAAAGGAGAACAGCTGAATG | |
| sacAmut2R | TACCTGGCTTGGTAGTGATTGAG | |
| agl4mutF | GTTTTAGAAACGCCAGTCTATGTG | |
| agl4mutR | ATTACACGTTAGATCAACTGGCAA | |
| sacR2mutF | GGTATACTACTGACAGCTTCCAAGG | |
| sacR2mutR | GCTGTTGCGAGTTTATTTTGTCT | |
| M13F-47 (FAM) | CGCCAGGGTTTTCCCAGTCACGAC | |
| M13R-48 | AGCGGATAACAATTTCACACAGGA | |
| **Primers for ChIP** | | |
| Flag- sacR1F | | ACAAGGAGATTTTAGCCATGGGATTACAAGGATGACGACGATAAGA |
| Flag- sacR1R | | CGGGGTACCGAATTCCTCGAGTTACGCGGTAAGGCCCTTG |
| Flag- sacR2F | | CATGCCATGGACGATTACAAGGATGACGACGATAAGACAATGAAACCAAAATTAAATGA |
| Flag- sacR2R | | CCGCTCGAGTACGATTAACTCCTTAGTTACGC |
| 403F | | GAAATACCCGTCTAAGGAATTG |
| 403R | | TGGTCATGAATTAGTCTCGGA |
| ChIP-pts1F | | GTTTCGAAGGTCCCCTTAACG |
| ChIP-pts1R | | TTGTTATTATTGGGTCTGTAAAGCG |
| ChIP-sacAF | | ATTTAATAATGCAAGCGCTTTACAG |
| ChIP-sacAR | | CATATCATGACGATGAGCTCCTTT |
| ChIP-agl4F | | GGTTACAACGACCTGAGTTAGCG |
| ChIP-agl4R | | CAATGCCATCGTTATTAGCATCTT |
| ChIP-sacR2F | | AATGGTGGACTAAATGTTTTAAGGG |
| ChIP-sacR2R | | TCTTAGCAACTGAAACACTAGGCC |

**Table S2.** Nucleotide sequences of oligonucleotides harboring the putative transcription factor binding sites (TFBSs) and mutated sites used for electrophoretic mobility shift assays (EMSAs).

| TFBS | Sequence | Mutated TFBS | Mutated sequencea |
| --- | --- | --- | --- |
| TFBSsacA TFBSpts1 | AATGTCAAACGATTGA TGTAAAGCGCTTGCAT | TFBSsacAMUT TFBSpts1MUT | **CTG**G**AT**A**GTTTGC**T**AG AAGATTTGACGGGAGG** |
| TFBSagl4 | AAGTGGAATCGATTCC | TFBSagl4MUT | **TCA**T**AT**A**GAAT**A**GGAA** |
| TFBSsacR2 | TGGAAACGATTCCAAA | TFBSsacR2MUT | **AATGTGTC**A**G**TC**GGGG** |

a Mutated nucleotide are marked with bold.
